# Transcutaneous cardiac pacing competency among junior residents undergoing an ACLS course: impact of a modified high fidelity manikin

**DOI:** 10.1186/s41077-018-0082-5

**Published:** 2018-12-07

**Authors:** Caroline Ranger, Marie-Rose Paradis, Judy Morris, Roger Perron, Pierre Drolet, Alexis Cournoyer, Jean Paquet, Arnaud Robitaille

**Affiliations:** 1 Department of Family Medicine and Emergency Medicine, Université de Montréal, Montréal, Canada; 2 Centre d’apprentissage des attitudes et des habiletés cliniques, Université de Montréal, Montréal, Canada; 3 Department of Anesthesiology and Centre d’apprentissage des attitudes et habiletés cliniques de l’Université de Montreal (CAAHC), Pavillon Roger-Gaudry, Université de Montréal, 2900, boul. Édouard-Montpetit, 8e étage, local N-805, Montréal, Québec H3T 1J4 Canada; 4 Hôpital Sacré-Cœur de Montréal, Montréal, Canada

**Keywords:** Transcutaneous pacing, Advanced cardiac life support, Simulation training, Simulation mannequin, Simulation fidelity

## Abstract

**Background:**

Transcutaneous cardiac pacing (TCP) is recommended to treat unstable bradycardia. Simulation might improve familiarity with this low-frequency procedure. Current mannequins fail to reproduce key features of TCP, limiting their usefulness. The objective of this study was to measure the impact of a modified high-fidelity mannequin on the ability of junior residents to achieve six critical tasks for successful TCP.

**Methods:**

First-year residents from various postgraduate programs taking an advanced cardiovascular life support (ACLS) course were enrolled two consecutive years (2015 and 2016). Both cohorts received the same standardized course content. An ALS simulator® mannequin was used to demonstrate and practice TCP during the bradycardia workshop of the first cohort (control cohort, 2015) and a modified high-fidelity mannequin that reproduces key features of TCP was used for the second cohort (intervention cohort, 2016). Participants were tested after training with a simulation scenario requiring TCP. Performances were graded based on six critical tasks. The primary outcome was the successful use of TCP, defined as having completed all tasks.

**Results:**

Eighteen participants in the intervention cohort completed all tasks during the simulation scenario compared to none in the control cohort (36 vs 0%, *p* < 0.001). Participants in the intervention cohort were more likely to recognize when pacing was inefficient (86 vs 12%), obtain ventricular capture (48 vs 2%), and check for a pulse rate to confirm capture (48 vs 0%).

**Conclusions:**

TCP is a difficult skill to master for junior residents. Training using a modified high-fidelity mannequin significantly improved their ability to establish TCP during a simulation scenario.

**Electronic supplementary material:**

The online version of this article (10.1186/s41077-018-0082-5) contains supplementary material, which is available to authorized users.

## Background

Unstable bradycardia is an uncommon occurrence with an estimated incidence of six out of 10,000 patients seeking care in the emergency room (ER) [1]. Transcutaneous cardiac pacing (TCP) is an effective treatment recommended by the 2010 American Heart Association (AHA) guidelines for unstable bradycardia [2, 3]. It can, however, be difficult to implement: pitfalls must be avoided, and assessment of ventricular capture is complicated [4–6]. This low frequency but high-stakes procedure may pose a challenge for junior clinicians unfamiliar with unstable bradycardia.

Since pacing situations are rare and heterogeneously observed in the clinical context, experience alone is generally insufficient to acquire and maintain competency in TCP. Simulation provides an opportunity to practice TCP in a context conducive to learning, and the 2015 American Heart Association guidelines state that the use of high-fidelity mannequins for ACLS training can be beneficial [7]. However, even the highest-fidelity mannequins available have significant shortcomings regarding simulation of TCP, which might limit their usefulness. The Two-Mannequin Model (TMM), a new mannequin that simultaneously exploits technical features of two commercially available mannequins, has been proposed to overcome some of the weaknesses of the available mannequins regarding TCP simulation [8]. It is however important to establish whether this higher-fidelity simulation of TCP indeed leads to better learning of TCP [9, 10]. 

The objective of this study was to evaluate the impact of teaching with the modified high-fidelity TMM on the learning of TCP by junior residents during an ACLS provider course. Their competence was judged by their ability to complete the tasks necessary for successful TCP in a simulation scenario and compared to a similar cohort having been taught with the lower-fidelity mannequin traditionally used for teaching, using the same assessment scenario.

## Methods

### Study design and setting

This cohort study was conducted at the Centre d’apprentissage des attitudes et habiletés cliniques (CAAHC), Université de Montréal’s medical simulation centre, in Canada, in July 2015 and July 2016. It was approved by the local Institutional Review Board for research in healthcare (15-051-CERES-D).

Every July, the CAAHC offers a mandatory 2-day ACLS provider course to over 200 first-year residents beginning their postgraduate training. The course complies with all rules and requirements of the Heart and Stroke Foundation and follows the most recent AHA guidelines [12]. Its first day consists of lectures, and its second day of six hands-on learning stations organized around the ACLS algorithms. Each station lasts 50 min, with one certified instructor supervising no more than six participants. Bradycardia management is covered in a lecture on day 1 and a hands-on station on day 2.

### Study population and participant selection

Two consecutive cohorts of junior residents during their first month of postgraduate medical training were used as a study population (July 2015 and July 2016). First-year residents from both cohorts with no prior residency experience were approached on day 1 of the course. The study was presented as evaluating a teaching method without providing any specific information about which method was being investigated. All recruited volunteers signed informed consent waivers and a confidentiality agreement prior to inclusion in the study. The first cohort was the control cohort and the second cohort the intervention cohort.

### ALS simulator® mannequin (Laerdal Medical, Stavanger, Norway)

The ALS simulator mannequin (Laerdal Medical, Stavanger, Norway) was used during the bradycardia workshops of the first cohort (Control—July 2015) to demonstrate and practice TCP. This mannequin reproduces some aspects of TCP, with the following notable exceptions:

- It does not allow the use of multifunction pads, requiring instead special connectors not used in clinical practice (which cannot be misplaced)
- It does not present muscular twitching with increasing TCP output as a real patient would
- It does not reproduce electrical artifacts that can appear on the ECG tracing during TCP, and that can be misinterpreted as ventricular capture because they have the same frequency as the rate set on the TCP [5, 6].

### Modified high-fidelity TMM

The modified high-fidelity TMM was used during the bradycardia workshops of the second cohort (Intervention—July 2016) to demonstrate and practice TCP. This custom-made mannequin combines a modified Human Patient Simulator mannequin (HPS®, CAE Healthcare, Montreal, Canada) with whom the participants interact in the simulation suite and a SimMan 3G® (Laerdal Medical, Stavanger, Norway) located in an adjacent room and used solely to generate the electrocardiogram (ECG) tracings seen by the participants (the participants never interact with the SimMan 3G®, they only see its ECG tracing which comes out of the HPS mannequin and is displayed on the monitor). Compared to the ALS simulator®, the TMM’s response to TCP more closely resembles that of a real patient [8]. The TMM:

- Allows the use of the same multifunction pads used clinically (which can be misplaced on the chest)
- Replicates the muscular twitching of the patient with increasing TCP output, which can mislead users into inferring that pacing is effective
- Reproduces the artifacts that often appear on the ECG with TCP, as the electric current traveling through the chest with each stimulation is picked up by the monitor; the presence of these artifacts, which are synchronous with pacing, may be mistaken for pacemaker-generated wide complex QRS, giving a false impression of ventricular capture; users must therefore distinguish these artifacts from true ventricular capture just as they would clinically on a real patient, by palpating a pulse and checking if it corresponds to the rate set on the TCP
- Allows simultaneous ECG monitoring on the TCP and on a clinical monitor
- Can mimic many other characteristics of real patients (i.e., talks, closes its eyes) [13]

Supplemental videos further illustrate differences between the two mannequins (see video, Additional file 1, for the ALS simulator®) (see video, Additional file 2, for the TMM).


Additional file 1:TCP simulation with the ALS simulator® mannequin. (WMV 45130 kb)



Additional file 2:TCP simulation with the Two Mannequin Model (TMM). (WMV 61318 kb)


### Intervention

Both cohorts received a similar bradycardia workshop: the objectives were the same and were standardized following ACLS guidelines. Furthermore, the workshops were given by the same six ACLS instructors, under the supervision of the same course director. No change in ACLS guidelines occurred between the two years of the study for the bradycardia algorithm.

The bradycardia workshop focused on the management of unstable bradycardia as described by the ACLS guidelines [12]. A HeartStart XL® monitor/defibrillator (Philips, Andover, MA) was used to demonstrate and then practice TCP. The lower-fidelity ALS simulator® mannequin (Laerdal Medical, Stavanger, Norway), the one customarily used for ACLS courses at the Université de Montréal simulation center, was used for the bradycardia workshops for the first cohort. The modified high-fidelity TMM was used for the bradycardia workshops for the second cohort. Because they were not required by the workshop objectives, some features of the TMM were not used during the training sessions (e.g., talking, eyes closing and opening). For both cohorts, the participants’ learning of TCP was assessed through their ability to complete six critical tasks during a high-fidelity simulation scenario involving a case of unstable bradycardia with complete atrioventricular block (see Appendix 1). This test was administered after the morning stations on day 2 of the course, which always included the bradycardia station. In order for the mannequin to replicate as closely as possible a real patient, the modified high-fidelity TMM was used, exploiting not only its TCP capability, but also its full potential for integration in a scenario reproducing a real clinical context. Indeed, the simulation technician controlling the TMM could answer participants’ questions as well as control the TMM to interact with the participants by reacting to their actions (telling the participants that the TCP was painful, for example). Since the participants from the control cohort may not have had prior exposure to such a mannequin, a short video of a simulation scenario demonstrating its features was presented to all participants during the first day of ACLS training. The exact same simulation scenario was repeated with every participant, regardless of their cohort. Great care was taken to ensure that participants and workshop instructors were not aware of the content of the scenario.

Before the simulation, each participant completed a survey about demographics and prior clinical and simulation experience. An individual briefing was then given about the context of the simulated case. During the briefing, participants were explicitly told to act as they would during a real patient encounter (e.g., talk to the patient and expect an answer, appropriately examine the patient). The participant was then invited into the simulation suite to begin the scenario, during which a facilitator acted as a nurse. Participants could interact with the patient (TMM), order any test, and start any treatment deemed necessary.

All simulations were video-recorded. Based on the work of Ahn et al. [4], the following six tasks were used to determine clinical competency in TCP (see Appendix 2):

1. Turning on pacer function within 4 min
2. Applying multifunction pads correctly
3. Recognizing that TCP is ineffective initially
4. Achieving ventricular capture
5. Verifying capture by taking the pulse
6. Prescribing sedation or analgesia

The time to completion of each task was also noted.

The scenario was allowed to run until all tasks had been completed, or up to a maximum of 9 min. For the first task only, a time limit of 4 min was used, after which the facilitator recommended TCP use. In this case, this task was considered as not having been done, but the scenario was allowed to continue. Scripted debriefing systematically followed simulation, after which participants completed a survey on their previous experience with bradycardia management and TCP.

Two investigators (CR, MRP) graded each participant’s performance using the video recording of the simulation. In case of discrepancy, a third investigator (AR) was involved and the results were determined by consensus. The time to completion of each task was evaluated using the same technique.

### Study outcomes

The primary outcome measure was the completion of all six previously mentioned tasks during the course of the simulation scenario. The secondary outcomes were the completion of each of the six tasks on an individual basis and the time to completion of each of the six tasks on an individual basis.

### Sample size calculation and statistical analyses

Based on previous experience running a simulation scenario similar to the one used in this study with 20 cohorts of ACLS-trained internal medicine residents, it was estimated that the success rate in our control cohort would be 20%. Including 50 participants per cohort would allow the detection of a 30% absolute difference regarding the success rate, accepting alpha and beta errors of 0.05 and 0.1 respectively. It was planned to exclude and replace candidates with a breach of protocol.

Continuous variables are presented as means with standard deviations and categorical variables are presented as frequencies with percentages. Cohort differences for demographic, prior clinical, and simulation experience were assessed using Student’s *t* test and Pearson’s chi-squared test, as appropriate.

For the primary outcome, the proportion of each cohort who completed all six tasks were compared using a Pearson’s chi-squared test. For the secondary outcomes, the proportion of each cohort who completed each individual task were described as the time to completion of each of these individual tasks. Statistical analyses were performed using SPSS Statistics 23.0 (IBM, Chicago, USA) and PRISM 7 (GraphPad, La Jolla, USA). Alpha levels were fixed at 0.05.

## Results

### Characteristics of the participants

One hundred and four participants were recruited in the study, 53 in the control cohort (2015) and 51 in the intervention cohort (2016). Two participants were excluded in 2015 due to prior participation in a residency program, and one participant was excluded each year due to the simulation scenario mistakenly ending earlier than 9 min. Fifty participants were therefore retained in each cohort. Table 1 shows the characteristics of the two cohorts. Study participants’ characteristics were similar in both cohorts, with the exception that more participants in the intervention cohort had previous exposure to high-fidelity simulation (26 vs 15, *p* = 0.05), while more participants in the control cohort had been exposed to a real case of unstable bradycardia (5 vs 0, *p* = 0.02). A majority of participants in both cohorts were beginning a family medicine residency, with the remainder starting various other residency programs (Table 2).

**Table 1:** Demographics and background of participants

Characteristics	Control cohort (*n* = 50)	Intervention cohort (*n* = 50)	*P* value
Age, years, mean (SD)	26 (3)	25 (2)	0.07
Male, *n* (%)	23 (46)	13 (26)	0.04
Family medicine residents, *n* (%)	29 (58)	28 (56)	0.99
Time since ACLS course, *n* (%)			0.77
No previous ACLS course	31 (62)	34 (68)	
< 12 months	6 (12)	6 (12)	
> 12 months	13 (26)	10 (20)	
Hours of preparation for the ACLS course, *n* (%)			0.88
0–4	30 (60)	29 (58)	
5–10	15 (30)	17 (34)	
> 10	5 (10)	4 (8)	
Previous simulation experience, *n* (%)			0.05
None	5 (10)	6 (12)	
Low-fidelity	30 (60)	18 (36)	
High-fidelity	15 (30)	26 (52)	
Previous clinical experience with unstable bradycardia, *n* (%)	5 (10)	0 (0)	0.02
Previous experience with TCP, *n* (%)	1 (2)	0 (0)	0.32

*ACLS* advanced cardiovascular life support; SD, standard deviation; TCP, transcutaneous pacing

**Table 2:** Participants training program

Training program	Control cohort (*n* = 50)	Intervention cohort (*n* = 50)
Family medicine	29	28
Medicine subspeciality (internal medicine, neurology, dermatology)	5	5
Surgical subspeciality (general surgery, orthopedics, vascular surgery, neurosurgery, obstetric/gynecology, ENT, urology)	5	5
Others	11	12
Diagnostic radiology	3	3
Emergency medicine	0	1
Laboratory medicine (microbiology, anatomy-pathology)	2	3
Ophtalmology	0	2
Pediatrics	1	0
Physical medicine and rehabilitation	1	3
Psychiatry	4	0

### Main results

**Fig. 1:**
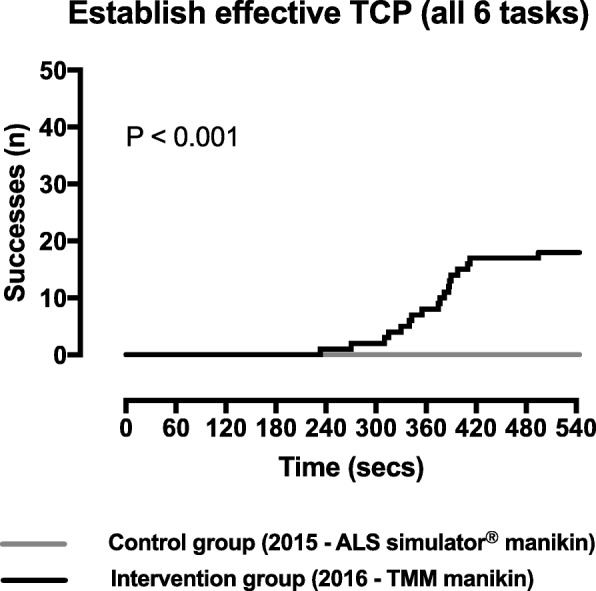
Cumulative incidence curves for establishing effective TCP during the simulation scenario (completing all six critical tasks)

In the intervention cohort, eighteen participants (36%) successfully completed all six tasks during the simulation scenario compared to no participants in the control cohort (*p* < 0.001) (Table 3 and Fig. 1).

**Table 3:** Participants performances in study scenario

	Control cohort (*n* = 50)	Intervention cohort (*n* = 50)	*P* value
Residents having successfully achieved TCP (completed all 6 tasks), *n* (%)	0 (0)	18 (36)	< 0.01
Residents having successfully completed each individual task			
1. Turning on pacer function within 4 min, *n* (%)	41 (82)	42 (84)	
2. Applying multifunction pads, *n* (%)	50 (100)	47 (94)	
3. Recognizing that TCP is ineffective, *n* (%)	6 (12)	37 (86)	
4. Achieving capture, *n* (%)	1 (2)	24 (48)	
5. Verifying mechanical capture, *n* (%)	0 (0)	24 (48)	
6. Prescribing sedation and/or analgesia, *n* (%)	45 (90)	43 (86)	

*TCP* transcutaneous pacing

**Fig. 2:**
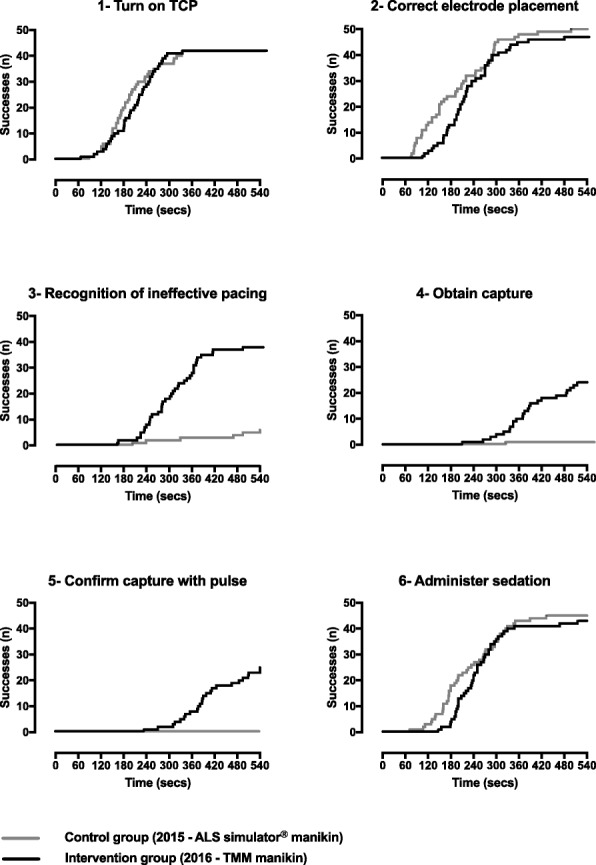
Cumulative incidence curves for each of the six critical tasks used to define effective TCP during the simulation scenario

Participants in the intervention cohort were more likely to recognize that pacing was inefficient initially (86%vs 12%), to obtain ventricular capture (48% vs 2%) and to check for a pulse (48% vs 0%) (Table 3). Additionally, they completed these three tasks more rapidly than participants in the control cohort (Fig. 2).

The proportion of participants that turned on the TCP function within 4 minutes was not different between the intervention and control cohorts (84 vs 82%), and there was not any important difference between the proportion that applied the multifunction pads correctly (94% vs 100%) and between those offering sedation or analgesia to the patient (86% vs 90%).

## Discussion

The objective of this study was to evaluate the impact of a modified high-fidelity TMM compared to lower-fidelity mannequins traditionally used in the learning of TCP. Learning TCP was difficult for junior residents, as demonstrated by their generally low success rate. However, the use of a modified high-fidelity TMM during an ACLS course, when compared to a traditional simulation mannequin, significantly improved their ability to complete all tasks necessary for successful TCP during a simulated case resembling clinical practice. This study contributes to the literature by demonstrating for the first time the ability of such a mannequin to improve the learning of TCP and for a specific intervention other than repeated practice to improve the ability to establish TCP.

These results are consistent with the broader literature which suggests that the use of high-fidelity mannequins for advanced life support (ALS) training is associated with improved skill performance at course conclusion [18]. However, with most studies measuring overall performance, it is difficult to understand exactly when improved fidelity leads to better performance [9]. Studies like this one, which focus on a specific skill and on specific characteristics of the simulated experience, can help to pinpoint where efforts to improve simulation fidelity are likely to lead to benefits.

The differences between the both cohorts’ results can be explained by multiple factors. Being deceived by intuitively appealing but false signs of ventricular capture upon initiating TCP is a recognized pitfall of the technique [5, 6]. Many participants in our study assumed ventricular capture upon seeing the mannequin twitch or upon noting on the ECG a higher-frequency signal of pacer spikes followed by wide QRS-like complexes. These participants either did not take a pulse despite having been instructed specifically to do so during training or took the pulse but did not appreciate it was still very slow. When questioned during debriefing, these residents frequently mentioned that they were so convinced the TCP was effective that they did not feel the need to take the pulse or to calculate its frequency, suggesting a confirmation bias in their processing of the clinical information [17]. Improved simulation in the second year of the study might have led to a better performance by allowing participants to experience and understand the false signs of ventricular capture during training. Interestingly, checking the pulse was also the step most often omitted in the study involving the deliberate practice of TCP in emergency medicine residents [4].

The use of a modified high-fidelity mannequin in the second year of this study might also have improved teaching by the instructors: more emphasis may have been put on false signs of effective TCP and on the importance of the pulse to confirm capture. A better TCP simulator might also have improved the instructors’ own understanding of TCP. If better teaching in the intervention cohort explains the superior results, it might be possible to achieve similar gains by improving teaching without using a high-fidelity simulation at all.

Although the modified high-fidelity TMM significantly improved the ability of junior residents to achieve all six tasks for successful TCP, a large proportion of residents were still unable to do so. Many explanations for the limited impact of ACLS training have been offered [14]. In this study, failure is likely attributable in part to the inexperience of our participants: first-year residents dealing with an unstable patient are expected to struggle both to rapidly extract and organize the pertinent clinical cues from the overwhelming amount of information provided by the realistic clinical setting and to respond correctly [15, 16].

Another explanation for the high failure rate in the present study might relate to the complexity of TCP. In a previous study involving more senior emergency medicine residents with some TCP experience, an average of three attempts were needed to successfully perform TCP on a simulator [4]. Furthermore, in that study, the attempts were made in a context of deliberate practice where feedback was given after each attempt, a setting more favorable than the one used to test participants in our study.

A suboptimal teaching method might also contribute to a high failure rate. Demonstrating and practicing a complicated technique on a simulator presenting an excessively simplified version of reality might lead to underperformance in more complex and more realistic situations. In the second year of this study, the use of an improved simulation tool during hands-on TCP training, in a similarly inexperienced population undergoing an otherwise identical course, led to a significant improvement in performance when measured in the same simulated clinical situation. More specifically, the intervention cohort outperformed the controls in three critical tasks simulated more effectively by the TMM. This supports the hypothesis that, in specific cases, increased fidelity can lead to better learning.

The use of simulation as an assessment tool is gaining acceptance [19, 20], and in this study, it provided a reliable way to measure TCP success. Furthermore, since the simulation experience recreated key physical, as well as the cognitive and emotional aspects of acute clinical care, this type of evaluation might be a more valid marker of actual clinical performance than the written tests and the “megacode” simulations traditionally used to assess ACLS competency.

The present study is not devoid of limitations. Some of the improvement in the intervention cohort might be attributable to participants becoming more familiar with the TMM mannequin used both during hands-on training and during simulation testing. To minimize this potential bias, a video illustrating the TMM’s general features was presented before the simulation scenario. The choice of a video instead of a hands-on orientation on the TMM for the control group prior to simulation testing was made because of time constraints and because of the risk that an orientation including TCP might either cue the participants about the contents of the simulation to come or serve as another learning opportunity, both factor potentially confounding our ability to assess what the participants really learned in the ACLS course. In addition, since the high-fidelity characteristics of the TMM used during training were only the ones relating to TCP, it is likely that any increased familiarity with the mannequin would relate chiefly to its response to pacing. If the simulator, despite its limitations [8], approaches clinical reality enough, then one can expect the gains in familiarity with pacing the TMM to transfer to pacing actual patients: the increase in familiarity and performance would represent a true gain in competence that would not be restricted solely to the TMM.

Another potential source of bias, given our study design, is that some participants might have known about the simulation scenario beforehand. However, all participants signed a confidentiality agreement and confirmed they had no knowledge of the scenario before beginning the project. Furthermore, participants had little incentive to share the information since they knew their performances were kept strictly confidential. The instructors might also have learned about the objectives of the study and modified their teaching method. However, great care was taken to prevent the instructors from knowing the content of the assessment scenario.

The use of junior residents limits the generalizability of our findings. While the homogenous recruitment population enabled the formation of comparable cohorts, this study’s findings might not apply to other populations of learners. Indeed, the appropriate level of fidelity to maximize learning is known to vary according to learner experience [11, 21]. This will remain a challenge with ACLS courses, which are offered to very heterogeneous populations of learners. Additionally, the junior residents were mostly family practice residents, and this may limit the applicability of our findings as TCP is less likely to be performed outside the ER. However, when it is performed outside the ER, it may be done so by clinicians less familiar with this procedure, as was the case in this study.

Finally, given the design of this study, it is only possible to speculate about how the participants’ performance translated in real-life situations, and it is not possible to know their long-term retention of TCP.

## Conclusions

The use of a modified high-fidelity mannequin during an ACLS course improves the ability of junior residents to achieve six critical tasks for successful TCP. However, an important performance gap remains and future research should aim to identify methods to address this gap [22].

## Appendix 1

### Simulation scenario and scoring

The participant is given the role of a junior resident on call in a general hospital and is initially requested by the nurse (facilitator) on the medicine ward for a bradycardic and hypotensive 80-year-old patient admitted for pneumonia. The nurse is already in the room when the participant enters, and announces that the patient has just vomited. The patient’s eyes are closed initially, but he is awake and alert enough to open his eyes when stimulated and to answer questions. His vital signs are blood pressure 70/40 mmHg, heart rate 35 beats per minute with a third-degree atrioventricular block, oxygen saturation 93% on room air. Vital signs are shown on a monitor to which the manikin is already attached. An oxygen mask and a crash cart equipped with a HeartStart XL® defibrillator/monitor with multifunction pads are available at the bedside.

The bradycardia is refractory to any medical treatment and remains symptomatic until a transcutaneous cardiac pacemaker (TCP) is efficiently installed. If the participant orders the administration of atropine, ephedrine or phenylephrine, the nurse announces, after a 10 s delay, that the drug does not work in order to reinforce the need for another therapy. If the participant orders the administration of dopamine, adrenaline, or noradrenaline, the nurse answers that these therapies will only be available upon arrival of the ICU team, which is on its way (but does not arrive during the 9 min duration of the scenario). If the participant asks for help or for a consultant, a call is made but the consultant does not call back and help does not arrive.

One minute into the simulation scenario, the plethysmography signal is lost. If the participant asks why, the nurse explains that the patient’s extremities are cold and that this can explain such a loss of signal. Four minutes into the scenario, if the participant is not actively engaged in setting up the TCP, the nurse suggests it and the participant is considered to have failed to achieve the *first critical task*. If the participant sets up the TCP and fails to recognize the need to apply multifunction pads or applies them incorrectly (i.e., other than in the anteroapical or anteroposterior positions), this leads to a failure of the *second critical task*. The pacing threshold is set arbitrarily high at 150 mA so that the participants are unlikely to set the output above the threshold initially, enabling raters to assess the *third critical task*, which is to recognize that TCP is ineffective initially. If the TCP output is set initially above the threshold, the facilitator emphasizes the patient’s discomfort and insists on decreasing the current. If the participant sets the current ≥ 150 mA and maintains it at that level, stable ventricular capture is achieved (*fourth critical task*): the patient improves clinically and blood pressure normalizes. The participant then needs to manually check the pulse to achieve the *fifth critical task* (because the plethysmography signal is lost after 1 min, the participant cannot use it to assess ventricular capture). As soon as the TCP is started, regardless of the output chosen, the patient starts to complain of pain periodically. The participant can prescribe sedation or analgesia at any moment during the scenario (*sixth critical task*).

The scenario is allowed to run for up to 9 min or until all six critical tasks are fulfilled, whichever occurs first. The participant is subsequently brought into the debriefing room where an 8 min scripted debriefing session takes place.

## Appendix 2

**Table 4:** Critical tasks and clinical significance

Criteria	Clinical significance
1. Turning on pacer function	The participant recognizes that TCP is indicated and is able to turn on the pacemaker.
2. Applying multifunction pads	The participant is able to properly apply the multifunction pads (anterolateral or anteroposterior position).
3. Recognizing that the TCP is ineffective	The participant is able to recognize that the TCP is ineffective, either because the initial intensity of the TCP is inadequate, the connection of the multifunction pads is incorrect, or because the pacing mode has not been properly activated.
4. Achieving capture	The participant obtains ventricular capture by pacing above the threshold (150 mA) and then maintains capture through time.
5. Verifying mechanical capture	The participant manually verifies the pulse rate of the mannequin and confirms that it corresponds to the pacing rate.
6. Prescribing sedation and/or analgesia	The participant prescribes medication to decrease the discomfort associated with TCP.

*TCP* transcutaneous pacing

## Abbreviations

ACLS: Advanced cardiovascular life support
ALS: Advanced life support
TCP: Transcutaneous cardiac pacing
TMM: Two-Mannequin Model
